# Long COVID: cognitive, balance, and retina manifestations

**DOI:** 10.3389/fmed.2024.1399145

**Published:** 2024-07-05

**Authors:** Meritxell Carmona-Cervelló, Brenda Biaani León-Gómez, Rosalia Dacosta-Aguayo, Noemí Lamonja-Vicente, Pilar Montero-Alía, Gemma Molist, Aitana Ayet, Carla Chacón, Anna Costa-Garrido, Victor M. López-Lifante, Valeria Zamora-Putin, Liudmila Liutsko, Rosa García-Sierra, Antònia Fornés, Eduard Moreno-Gabriel, Marta Massanella, Jose A. Muñoz-Moreno, M. Carmen Rodríguez-Pérez, Lourdes Mateu, Anna Prats, Maria Mataró, Marc Boigues, Bibiana Quirant, Julia G. Prado, Eva Martínez-Cáceres, Concepción Violán, Pere Torán-Monserrat, the APC Collaborative Group

**Affiliations:** ^1^Unitat de Suport a la Recerca Metropolitana Nord, Institut Universitari d'Investigació en Atenció Primària Jordi Gol (IDIAP Jordi Gol), Mataró, Spain; ^2^Germans Trias i Pujol Research Institute (IGTP), Badalona, Spain; ^3^Grup de Recerca en Impacte de les Malalties Cròniques i les Seves Trajectòries (GRIMTra), Institut Universitari d'Investigació en Atenció Primària Jordi Gol (IDIAPJGol), Barcelona, Spain; ^4^Multidisciplinary Research Group in Health and Society (GREMSAS), Institut Universitari d'Investigació en Atenció Primària Jordi Gol (IDIAPJGol), Barcelona, Spain; ^5^Faculty of Medicine, University of Vic-Central University of Vic, Barcelona, Spain; ^6^Palau-Solità Healthcare Centre, Institut Català de la Salut, Palau-Solità Plegamans, Barcelona, Spain; ^7^Department of Medicine, Universitat Autònoma de Barcelona, Barcelona, Spain; ^8^Nursing Department, Faculty of Medicine, Universitat Autònoma de Barcelona, Barcelona, Spain; ^9^Department of Social Psychology, Universitat Autònoma de Barcelona, Barcelona, Spain; ^10^IrsiCaixa-AIDS Research Institute, Can Ruti Campus, Badalona, Spain; ^11^Centro de Investigación Biomédica en Red de Enfermedades Infecciosas (CIBERINFEC), Instituto de Salud Carlos III (ISCIII), Madrid, Spain; ^12^Red Española de investigación en Covid Persisitente, Barcelona, Spain; ^13^Infectious Diseases Department, Fundació Lluita Contra les Infeccions, Germans Trias i Pujol Hospital, Barcelona, Spain; ^14^Faculty of Psychology and Education Sciences, Universitat Oberta de Catalunya, Barcelona, Spain; ^15^Department of Clinical Psychology and Psychobiology, University of Barcelona, Barcelona, Spain; ^16^Institut de Neurociències, University of Barcelona, Barcelona, Spain; ^17^Institut de Recerca Sant Joan de Déu, Barcelona, Spain; ^18^Immunology Department, FOCIS Center of Excellence, Universitat Autònoma de Barcelona, Barcelona, Spain; ^19^Immunology Division, Laboratori Clínic Metropolitana Nord, Hospital Universitari Germans Trias i Pujol, Barcelona, Spain; ^20^Department of Cellular Biology, Physiology and Immunology,Universitat Autònoma de Barcelona, Bellaterra, Barcelona, Spain; ^21^Red de Investigación en Cronicidad, Atención Primaria y Prevención y Promoción de la Salud, Instituto de Salud Carlos III, Madrid, Spain; ^22^Department of Medicine, Faculty of Medicine,Universitat de Girona, Girona, Spain

**Keywords:** long COVID, neurological symptoms, neuropsychological assessment, postural balance, retina fundus

## Abstract

**Background:**

The neurological symptoms of Long COVID (LC) and the impact of neuropsychological manifestations on people’s daily lives have been extensively described. Although a large body of literature describes symptoms, validating this with objective measures is important. This study aims to identify and describe the effects of Long COVID on cognition, balance, and the retinal fundus, and determine whether the duration of symptoms influences cognitive impairment.

**Methods:**

This cross-sectional study involved LC volunteers with cognitive complaint from public health centers in northern Barcelona who participated between January 2022 and March 2023. This study collected sociodemographic characteristics, information on substance use, comorbidities, and clinical data related to COVID-19. We measured five cognitive domains using a battery of neuropsychological tests. Balance was assessed through posturography and retinal vascular involvement by retinography.

**Results:**

A total of 166 people with LC and cognitive complaints participated, 80.72% were women and mean age was 49.28 ± 8.39 years. The most common self-reported symptoms were concentration and memory deficit (98.80%), brain fog (82.53%) and insomnia (71.17%). The 68.67% presented cognitive deficit in at least one domain, with executive functions being the most frequent (43.98%). The 51.52% of the participants exhibited a dysfunctional pattern in balance, and 9.2% showed some alteration in the retina. There were no statistically significant differences between cognitive impairment and symptom duration.

**Conclusion:**

Our findings contribute to a more comprehensive understanding of the pathology associated with Long COVID. They highlight the diversity of self-reported symptoms, the presence of abnormal balance patterns, and some cognitive impairment. These findings underscore the necessity of addressing the clinical management of this condition in primary care through follow-up and the pursuit of multidisciplinary and comprehensive treatment.

## 1 Introduction

Most people who became infected with COVID-19 recovered completely, but approximately 3 to 30% might experience a variety of medium-term to long-term effects after the initial illness (1–3). Post COVID-19 condition, also known as Long COVID (LC), it described by the World Health Organization (WHO) as the persistence or emergence of symptoms 3 months after SARS-CoV-2 infection that persist for at least2 months and cannot be explained by an alternative diagnosis (4). LC can affect anyone exposed to the SARS-CoV-2 virus, regardless of the clinical spectrum of the acute illness or age (5).

Some studies posit that SARS-CoV-2 infection may result in endothelial damage through a pro-inflamatory cytokine storm, oxidative stress, coagulation imbalance, and immune cell response, ultimately leading to chronic low-grade inflammation (6, 7). This can caused a non-specific systemic constellation of persistent symptoms involving different organ systems, including neurological, vascular, musculoskeletal, respiratory and others (8). Recent evidence suggest that the most frequent neuropsychological manifestations are fatigue, brain fog, cognitive decline, sleep disturbances, and anxiety (9, 10). Some symptoms may persist for years (11, 12), and it is unclear if they can be established for life (13). The characteristics significantly impact the individual work performance (14), psychosocial well-being and quality of life (15). In addition, it imposes a burden on the health system (16), economy, and social spheres.

Cognitive sequelae are among the most disabling neurological symptoms that affect a high proportion of people with LC. A meta-analysis of LC patients reported that about 32% suffered from brain fog, 28% had memory disturbances, and 22% had attentional difficulties (17). Many studies that evaluated cognition found widespread cognitive impairment (18, 19). Moreover, imaging studies revealed structural and functional changes associated with cognitive assessments scores due to SARS-CoV-2 infection in the brain (20, 21). Additional research effort are needed to understand neurocognitive function in LC by adopting domain-specific assessment tools.

People with LC often experience ontological/vestibular symptoms such as dizziness, vertigo, and tinnitus (22). It appears that the SARS-CoV-2 virus can affect the systems related to balance (23–25). However, current studies are based on subjective methods such as questionnaires or case reports. Alternative, posturographic tests are an objective assessment to measure balance alterations.

Considering the endothelial dysfunction hypothesis, several reports have shown signs of vascular disorders in different organ systems due to COVID-19. The virus can affect the endothelium through the angiotensin-converting enzyme 2 (26) and cause direct damage to the vascular endothelial cells, and it is possible to detect it in the retina. Therefore, retinal examination by retinography, a valuable tool for studying the clinical effects of COVID-19 *in vivo*.

The persistence and consequences of LC underscore the need to delineate the areas of involvement and associated factors to formulate enhancements in the therapeutic interventions for individuals with this condition. Therefore, it is important to understand how LC affects cognition, balance, and ocular health. This study examines the cognitive, balance and retinal outcomes and explores the relationship between the duration of LC symptoms and the degree of neurocognitive impairment.

## 2 Methods

### 2.1 Study design and participants

This study is part of the Aliança ProHEpiC Cognitiu (APC) project, which aims to characterize the alterations in people with LC. More details regarding the project can be found in the published study protocol (27). This article presents the results of participants with LC and cognitive complaints.

The inclusion criteria were: (a) confirmed diagnosis of LC according to WHO criteria, (b) at least 12 weeks after infection (c) with cognitive complaints and (d) age between 18 and 70 years. The exclusion criteria were: (a) established diagnosis before COVID-19 infection of psychiatric, neurological, neurodevelopmental disorder pathologies known to cause cognitive deficits, (b) inability to perform neuropsychological examination due to literacy or sensory impairment, (c) history of illicit drug use, defined as habitual drug use (more than once a week) for at least 1 year or sporadic use (more than once a month) in the last 5 years, (d) alcohol abuse defined in accordance with the Spanish Ministry of Health risk consumption guidelines (28) (more than 20 gm/day in men or 10 gm/day in women) on a habitual basis for a period longer than 1 year, (e) medical conditions that limit participation and follow-up in the study (e.g., terminal illness).

### 2.2 Procedure

**Figure 1:**
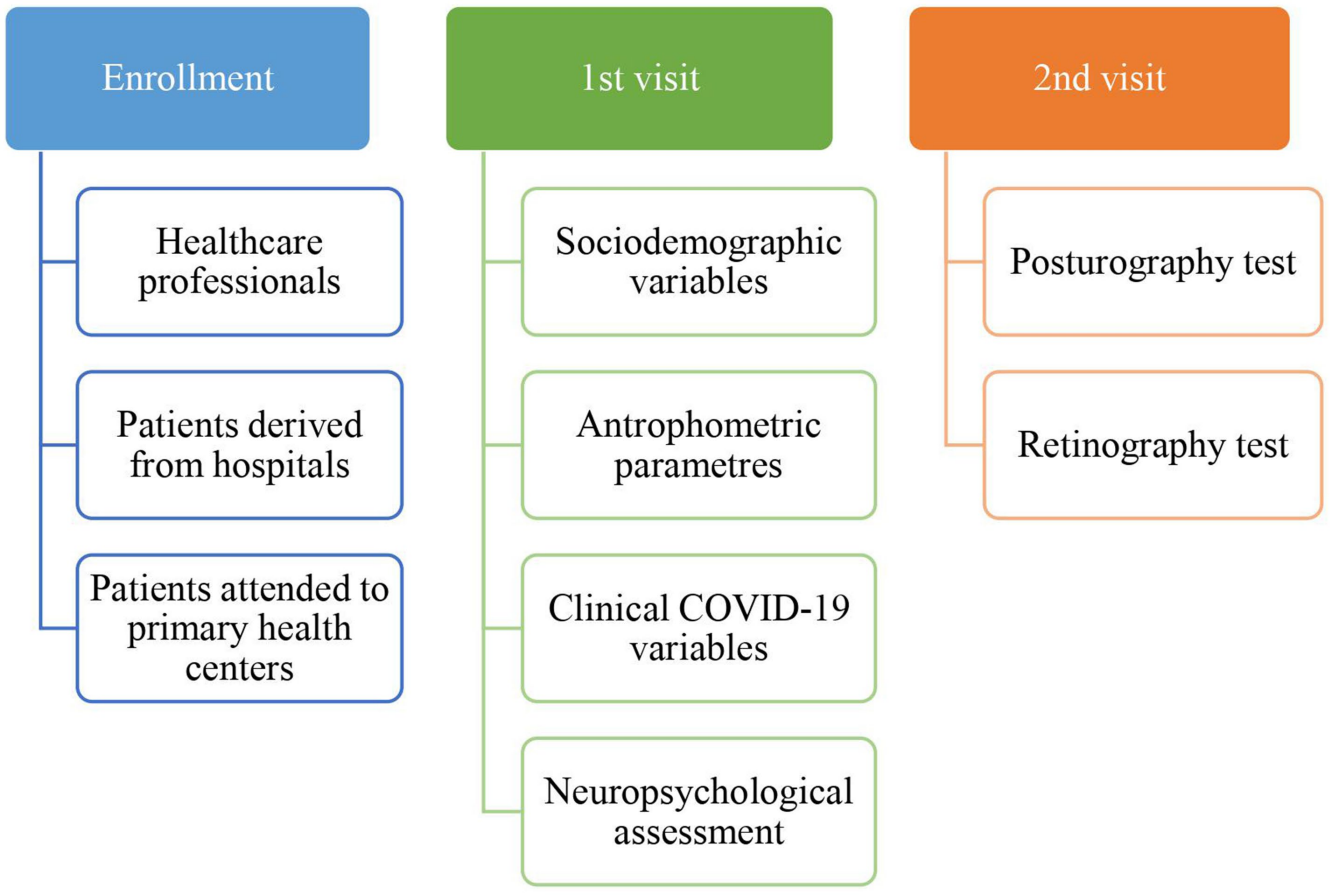
Diagram of the study design and the information collected at each visit.

Clinical and epidemiological characteristics were collected on two visits. During the baseline visit, participants provided sociodemographic information, anthropometric parameters, and vascular risk factors such as substance abuse and comorbidities, and were asked about their COVID-19 experience. Finally, all participants completed a comprehensive neuropsychological assessment. During the second visit, the balance capacity was measured using the posturography test, and eye fundus was explored using retinography (see Figure 1).

### 2.3 Variables

#### 2.3.1 Demographical, anthropometrical, and clinical variables

Demographics such as sex (women, man), age labeled as (20–34, 35–44, 45–54, 55–70), educational level (primary, secondary, high School, university degree, specialist or master, doctorate) and job field (medical doctor, nurse, health services, health assistants and others) were collected.

Anthropometric and clinical baseline measures weight (kg), height (cm), body mass index (according to the WHO standards (29)), high blood pressure, high cholesterol, diabetes, tobacco and alcohol consumption and frequency (times per day) were collected.

#### 2.3.2 Clinical COVID-19 variables

Diagnosis of SARS-CoV-2 infection variables were collected as date and methods of diagnosis (polymerase chain reaction, rapid antigen test, serology, and symptoms), and severity of symptoms (asymptomatic, mild/moderate, admission to hospital, admission to intensive care unit).

LC symptoms self-reported and duration were collected, labeled as (a) non-cognitive neurological symptoms: migraine, cephalalgia, non-specific polyneuropathy, myopathy, neuralgia and neuritis, cutaneous sensitivity alteration, cutaneous paresthesia, other cutaneous sensitivities, non-specific cutaneous sensitivity, altered consciousness, vertigo and dizziness and non-specific insomnia; (b) cognitive neurological symptoms: nonspecific disorientation, retrograde amnesia, other amnesia, other cognitive, dyslexia and symbolic disturbances, brain fog and lack of concentration and memory; (c) no neurological symptoms: cardiologic, skin, digestive, general, ocular, otorhinolaryngology, pulmonary, rheumatic, urologic and hormonal (see Appendix 1).

We collected variables related to the treatment of LC symptoms, categorized in pharmacological (antidepressants, anxiolytics, others) or non-pharmacological (cognitive training, yoga, reiki, acupuncture, bach flowers, prescribed physical exercise, others).

#### 2.3.3 Neuropsychological variables

All participants underwent a comprehensive neuropsychological battery conducted by a certified neuropsychologist. Five cognitive domains were evaluated: (a) executive functions (b) attention and processing speed, (c) memory, (d) visuospatial and visuoconstructive functions, and (e) language (see Table 1). The tests were selected based on expert consensus and considering the recommendations of the NeuroCOVID International Neuropsychology Taskforce (30). The participants’ raw test scores were standardized to Z-scores based on their age and years of education. The Z-scores range from −3 to 3, with 0 representing the mean. The Z-score indicate the extent to which a raw score deviates from the mean in standard deviation units.

**Table 1:** Description of cognitive domains assessed and neuropsychological tests administered.

Domain	Subdomain	Neuropsychological test
Executive functions	Working memory	Digit span backward (WAIS-III)
		TMT B - A (time)
	Verbal fluency	Phonetic fluency (PMR)
		Semantic fluency (animals)
	Inhibition	Stroop word-colors (interference)
Attention and processing speed	Attention	Digit span forward (WAIS-III)
	Processing speed	SDMT (WAIS-III)
		TMT A (time)
		Symbol search (WAIS-III)
Memory	Verbal memory	RAVLT (summarize)
		RAVLT (delayed recall)
	Visual memory	ROCF (delayed recall)
Visuospatial and visuoconstructive functions	Visuospatial and visuoconstructive	ROCF (copy accuracy)
		Language	Language	BNT Vocabulary (WAIS-III)

WAIS-III, Wechsler Adult Intelligent Scale third edition. TMT, Trail Making Test (part A and B). SDMT, Symbol Digit Modalities Test. RAVLT, Rey Auditory Verbal Learning Test. ROCF, Rey-Osterrieth Complex Figure. BNT, Boston Naming Test.

The tests used to evaluate the subdomains of executive functions were the time difference between parts B and A of the Trail Making Test (TMT) (31, 32) and the Digit Span Backward subtest from the Wechsler Adult Intelligent Scale (WAIS-III) (33) for the working memory. The verbal fluency was assessed by the number of words beginning with the letters P, M and R and the category “animals” (31, 34) recalled in one minute. The interference score of the Stroop test color-words was calculated as a measure of cognitive inhibitory control (35). The Digit Span Forward subtest (WAIS-III) was administered to measure attention (33). Visual scanning and motor speed were assessed by part A of the TMT (31, 32), Symbol Digit Modalities Test (SDMT) and Symbol Search from the WAIS-III (33). We used the Spanish version of Rey’s Auditory Verbal Test (RAVLT) (36) for verbal memory and visual memory was evaluated with the 30-min delayed recall test from the Rey-Osterrieth Complex Figure Test (ROCF) (31, 37). The copy accuracy of the ROCF was used to assess visuospatial and visuoconstructive abilities. The Spanish short version (15-items) of the Boston Naming Test (BNT) (38) and vocabulary subtest from the WAIS-III (33) were used to evaluate language.

#### 2.3.4 Posturography variables

For posturography, a dynamometric platform (Dinascan/IBVP600) was used to evaluate gait, gait speed and balance by a trained technician. The Romberg’s test (ROA, ROC, RGA, RGC) was used to evaluate postural control with more than two repetitions in each test. Each parameter expresses the percentage value of the variation with respect to the normality. Relation with different types of Romberg’s test automatically provided three indices (somatosensory, vestibular, and visual). The information from the indexes has been used to establish equilibrium patterns following an expert clinical consensus. For detailed information see Appendix 2.

#### 2.3.5 Retinography variables

To assess the eye fundus, a Topcon (TRC-NW8) with a non-mydriatic retinal camera was used by a trained technician to obtain entire central, nasal, and temporal retina images from both eyes. The images were anonymized and placed in the same position on the screen with a 16.2-megapixel resolution and a 45° field of view. High-quality control was applied to detect and eliminate images with poor resolution. A trained medical doctor conducted clinical image analysis manually; cases with detected abnormalities were referred to an ophthalmologist.

### 2.4 Statistical analysis

Categorical variables were described by each frequency and percentage. Continuous variables were described by mean, standard deviation and range. Descriptive analysis was used to characterize the sample sociodemographically and clinically. According to Frascati Criteria (39), an international consensus that has proved usefulness and reliability in another infection research field (40), we considered a cognitive deficit if one of the subtests was below −1.5 SD or if two subtests of the same cognitive domain were − 1 SD below the mean. Participants were classified as cognitively impaired if they had a deficit in at least two cognitive domains.

Subjects were classified into two groups according to the duration of the three symptoms previously defined: (a) 1^st^ group (G1) = 1 to 25 months of symptomatology and (b) 2nd group (G2) = 26 to 36 months symptomatology. *Post-hoc* analysis was carried out to compare the basal characteristics of G1 and G2 groups. Normality distribution of the data was tested with a Shapiro–Wilk test prior to each analysis. Time differences in demographic characteristics were analyzed as follows: independent 2-sample t-tests for normally distributed continuous variables, Mann–Whitney U-test for non-normally distributed continuous variables, and chi-square tests for categorical variables. All tests were two-sided, and a statistical probability of *p* < 0.05 was considered significant. Statistical analyses were performed using STATA Statistical Software (version 15.0; Statistical software for data science).

## 3 Results

### 3.1 Demographical, anthropometrical, and clinical variables

#### 3.1.1 Participants’ characteristics

A total of 182 participants were invited to participate in the study, 13 (7.14%) were excluded because they had a previous diagnosis associated with some type of cognitive impairment (attention deficit hyperactivity disorder, low intelligence quotient, previous stroke, language barrier and possibility of malingering) and three (1.64%) decided to abandon the study for different reasons (lack of availability and inability to contact).

Table 2 shows the sociodemographic characteristics of the 166 participants with LC and cognitive complaints included in the study. The 80.72% of the sample were women, with a median age of 49.28 years ±8.39 (range 25.5–69.8), and 39.76% had a job in the health services.

**Table 2:** Descriptive of the main characteristics of participants who present LC with cognitive complaints (*n* = 166).

Variable	n	(%)
Sex		
Women	134	(80.72)
Man	32	(19.27)
Age		
20–34	7	(4.22)
35–44	43	(25.90)
45–54	72	(43.37)
55–70	44	(26.51)
Educational level		
Primary	9	(5.42)
Secondary	7	(4.22)
High school	66	(39.76)
University Degree	66	(39.76)
Specialist / Master	16	(9.64)
Doctorate	2	(1.20)
Job field		
Doctor	10	(6.02)
Nurse	28	(16.87)
Health Services	10	(6.02)
Health Assistants	17	(10.24)
Others	101	(60.84)
Vascular Risk		
Hypertension	33	(19.88)
High Cholesterol	39	(23.49)
Diabetes	5	(3.01)
Alcohol	62	(37.58)
Smoking^a^	76	(46.06)
BMI^b^		
Underweight	6	(3.64)
Normal weight	57	(34.55)
Overweight	54	(32.73)
Obesity class I	23	(13.94)
Obesity class II	17	(10.30)
Obesity class III	8	(4.85)
Times diagnostic COVID-19		
1	80	(48.19)
2	68	(40.96)
3	11	(6.63)
4	7	(4.22)
Clinical spectrum COVID-19^c^		
Asymptomatic	2	(1.20)
Mild–Moderate	126	(75.90)
Hospitalization	34	(20.48)
ICU	4	(2.41)

BMI, Body Mass Index. ICU, Intensive care unit. All variables were self-reported, with the exception of BMI, which was measured during the baseline visit. ^a^The smoking category includes smokers and ex-smokers. ^b^According to WHO standards (16). ^c^Clinical spectrum variable refers to the first time of SARS-CoV-2 infection. The category “Mild–Moderate” encompasses any symptom manifestation that did not require medical attention.

#### 3.1.2 Clinical COVID-19 variables

Most participants (75.90%) had mild or moderate COVID-19 symptoms during their first infection, and more than half (51.81%) experienced reinfection. The most common neurological symptom reported was insomnia (71.17%), vertigo and dizziness (67.07%). All of them reported cognitive impairment, especially lack of concentration and memory (98.80%), followed by brain fog (82.53%). Almost the entirety of the sample exhibited some general symptoms, with asthenia being the most prevalent (42.11%) and musculoskeletal symptoms such as myalgia (70.12%). Some clinical variables had missing values: non-cognitive neurological symptoms (1.38%), cognitive neurological symptoms (1.03%), and no neurological symptoms (2.23%). Table 3 and Appendix 3 show the details of the symptoms reported by participants with LC.

**Table 3:** Symptoms self-reported and months duration at the time of assessment (*n* = 166).

	Total		Duration (months)^a^		
Symptoms	*n*	(%)	Mean [SD]	Min	Max
Non-cognitive neurological					
Altered consciousness	6	(3.68)	7.25 [11.21]	1	24
Cephalalgia	62	(37.58)	21.75 [8.71]	1	35
Cutaneous paresthesia	106	(65.03)	23.02 [8.49]	2	36
Cutaneous sensitivity	36	(22.09)	22.23 [8.86]	4	33
Hyperesthesia	31	(19.25)	24.9 [6.56]	7	33
Migraine	78	(46.99)	21.87 [8.26]	1	33
Myopathy	12	(7.27)	20.36 [10.49]	4	33
Neuralgia and neuritis	37	(22.70)	22.79 [7.82]	1	33
Nonspecific insomnia	116	(71.17)	23.42 [7.74]	1	36
Nonspecific polyneuropathy	23	(14.02)	22.53 [9.26]	1	31
Nonspecific sensitivity cutaneous	1	(0.61)	6	6	6
Other sensitivities cutaneous	2	(1.22)	15	15	15
Vascular cephalalgia	1	(0.61)	29	29	29
Vertigo and dizziness	110	(67.07)	22.48 [9.01]	1	36
Cognitive neurological					
Brain fog	137	(82.53)	22.72 [8.07]	3	36
Dyslexia and symbolic disturbances	21	(12.96)	20.61 [7.69]	4	31
Lack of concentration and memory	164	(98.80)	23.22 [7.41]	3	36
Nonspecific disorientation	75	(45.40)	21.23 [10.36]	2	35
Other amnesia	7	(4.29)	15.83 [9.11]	1	26
Other cognitive	91	(55.49)	23.86 [6.78]	4	36
Retrograde amnesia	3	(1.83)	19.67 [16.29]	1	31
No neurological^b^					
Cardiologic	76	(46.34)			
Digestive	96	(58.90)			
General	152	(92.68)			
Hormonal	42	(25.61)			
Ocular	61	(37.42)			
ORL	100	(61.35)			
Pulmonary	86	(52.76)			
Rheumatic	122	(74.39)			
Urologic	33	(20.37)			
Skin	70	(45.75)			

SD, Standard Deviation. Min, Minimum. Max, Maximum. ORL, Otorhinolaryngology. The symptoms self-reported and duration had several missing values. The symptoms that did not present any case are not shown in the table. ^a^No neurological symptoms duration was not collected. ^b^No neurological symptoms type is described in Appendix 3.

### 3.2 Neuropsychological, posturography, and retinography measures

Using the Frascati criteria (39) to assess the neuropsychological test battery results, we found that 52 participants (31.33%) in the sample were classified as cognitively intact, while 114 (68.67%) had a cognitive deficit in at least one domain. A total of 31.93% presented cognitive impairment with two or more domains affected (Table 4). The most frequently impaired cognitive domain was executive function (43.98%), followed by attention and processing speed (36.75%), and memory (28.31%) (Table 5). No significant associations were identified between the descriptive variables and cognitive impairment.

**Table 4:** Results of neuropsychological test, posturography, and retinography in people suffering from LC (*n* = 166).

Clinical assessment	*n*	(%)
Neuropsychological test^a^		
Intact	52	(31.33)
One domain	61	(36.75)
Two domains	36	(21.69)
Three domains	15	(9.04)
Four domains	2	(1.20)
Five domains	0	(0)
Posturography^b^		
Normal or compensated	75	(45.45)
Somatosensory dysfunction	20	(12.12)
Vestibular dysfunction	19	(11.52)
Visual dysfunction	4	(2.42)
Somatosensory dependence	7	(4.24)
Vestibular dependence	5	(2.42)
Visual dependence	16	(9.70)
Multisensory dysfunction	15	(9.09)
No assessable	5	(3.03)
Retinography		
Normal	151	(92.07)
Alteration	12	(7.54)
Hard exudates	8	(66.67)
Hemorrhages	4	(33.33)
Vascular occlusions	0	(0)
Venous dilatation	0	(0)
No assessable	1	(0.61)

^a^Cognitive impairment is defined as the presence of two or more deficits in different cognitive domains. ^b^The parameters utilized to ascertain the balance patterns are delineated in Appendix 2.

**Table 5:** Percentage for each test according to −1.0 SD and − 1.5 SD and Frascati Criteria.

	Cutoff −1.0 SD	Cutoff −1.5 SD	Frascati Criteria	Z-score^a^
Domain	*n* (%)	*n* (%)	*n* (%)	Mean [SD]
Executive functions			73 (43.98)	
Digit span backwards (WAIS-III)	9 (5.42)	6 (3.61)		−0.23 [0.80]
TMT B – A (time)	10 (6.02)	5 (3.01)		0.06 [0.74]
Phonetic verbal fluency (P)	33 (19.88)	20 (12.05)		−0.36 [0.92]
Phonetic verbal fluency (M)	18 (10.84)	11 (6.63)		−0.31 [0.86]
Phonetic verbal fluency (R)	25 (15.06)	16 (9.64)		−0.44 [0.82]
Semantic verbal fluency (animals)	70 (42.17)	49 (29.52)		−0.98 [0.93]
Stroop word-colors (interference)	7 (4.22)	3 (1.81)		0.35 [0.80]
Attention and processing speed			61 (36.75)	
Digit span forward (WAIS-III)	42 (25.30)	34 (20.48)		−0.51 [1.00]
SDMT (WAIS-III)	8 (4.82)	4 (2.41)		0.20 [0.79]
TMT A (time)	47 (28.31)	32 (19.28)		−0.72 [0.99]
Symbol Search (WAIS-III)	16 (9.64)	8 (4.82)		−0.02 [0.89]
Memory			47 (28.31)	
RAVLT (summarize)	60 (36.14)	30 (18.07)		−0.48 [1.20]
RAVLT (delayed recall)	33 (19.88)	16 (9.64)		−0.21 [1.00]
ROCF (delayed recall)	19 (11.45)	9 (5.42)		−0.40 [0.72]
Visuospatial and visuoconstructive functions			5 (3.01)	
ROCF (copy accuracy)	9 (5.42)	3 (1.81)		−0.12 [0.83]
Language			2 (1.20)	
BNT	0 (0)	0 (0)		1.53 [0.92]
Vocabulary (WAIS-III)	5 (3.01)	2 (1.20)		0.13 [0.62]

SD, Standard Deviation. WAIS-III, Wechsler Adult Intelligent Scale third edition. TMT, Trail Making Test (part A and B). SDMT, Symbol Digit Modalities Test. RAVLT, Rey Auditory Verbal Learning Test. ROCF, Rey-Osterrieth Complex Figure. BNT, Boston Naming Test. ^a^Data are presented as Z-scores. Negative Z-scores signifies that observation is below the mean value, whereas a positive Z-scores indicates that it is above the mean. Mean and standard deviation for each test expressed in Z-scores (*n* = 166).

The posturography test shows that 75 (45.45%) participants present a normal or compensated pattern. The more frequent patterns were somatosensory dysfunction (12.12%) and vestibular dysfunction (11.52%). Five people (3.03%) could not be evaluated because they were too exhausted to finish the test (Table 4). No significant associations were identified between the descriptive variables and balance patterns.

The 92.07% of individuals did not manifest any type of alteration in the retinography, 12 participants (7.54%) had visible affection in the ocular fundus (Table 4). The alteration found in at least one of the eyes was hard exudates (4.88%) and hemorrhages (2.44%). No significant associations were identified between the descriptive variables and retinal alterations.

### 3.3 Association of cognitive impairment and symptoms duration

**Figure 2:**
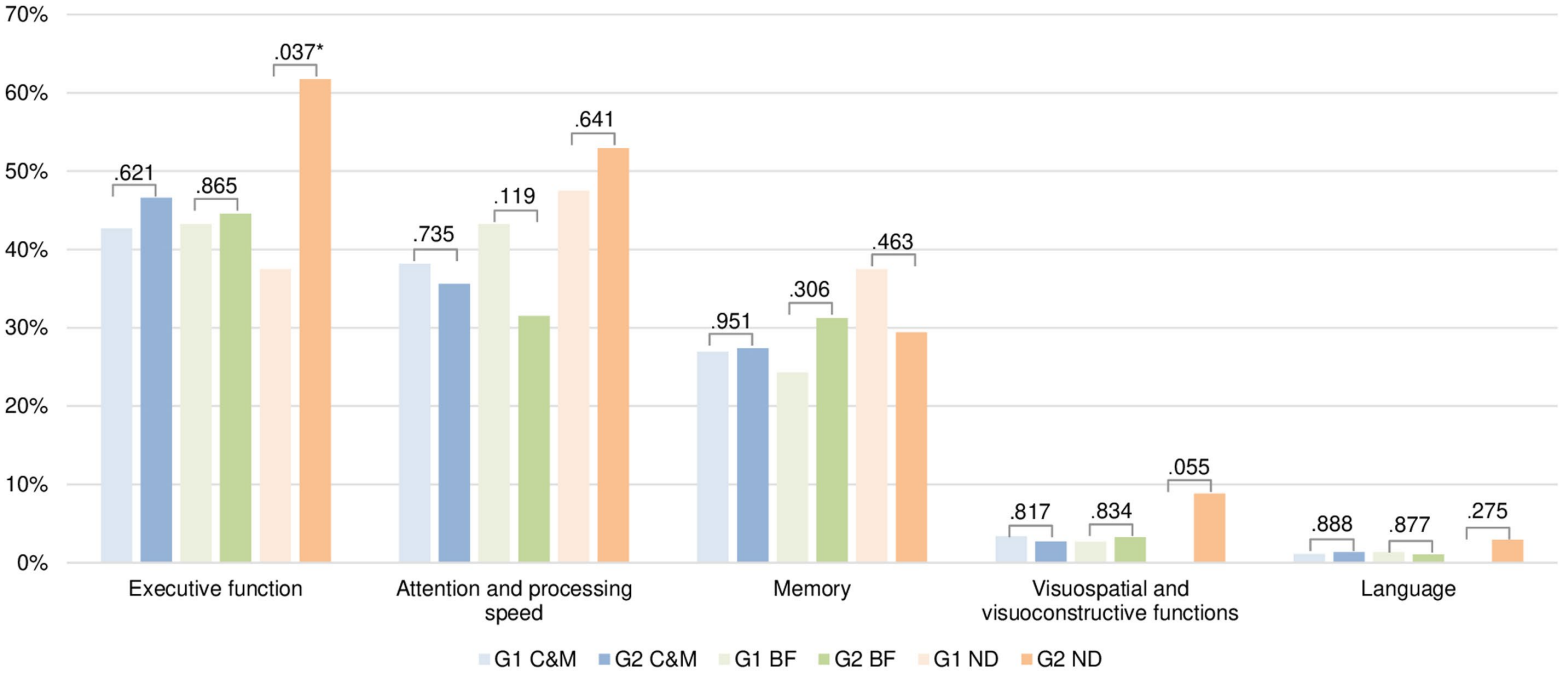
Differences between duration of cognitive symptoms self-reported (lack of concentration and memory, brain fog and nonspecific disorientation) and cognitive deficit by domain (*n* = 166).C&M, lack of Concentration and Memory. BF, Brain Fog. ND, Nonspecific Disorientation. Note: Symptom duration was divided into two groups: G1 (1 to 25 months) and G2 (26 to 36 months). The Figure shows the *p*-value of Chi-square analysis. We selected the most prevalent cognitive symptoms in our sample excluding the “Other cognitive symptoms” because it grouped more than one symptom.

Subjects were divided into two groups (G1 and G2) according to the duration of the most predominant cognitive symptoms reported: (a) lack of Concentration and Memory (C&M), (b) Brain Fog (BF) and (c) Nonspecific Disorientation (ND). There were no significant differences in demographic, anthropometric and clinical variables between these groups (see Appendix 4). Figure 2 shows the frequency of cognitive domain deficit by symptom duration. In the executive function domain, the group with a shorter duration of the three symptoms had better scores, with only the ND symptom showing a statistically significant difference (G1 = 37.50% vs. G2 = 61.76%, *p* = 0.037). There were no significant differences between the groups in terms of the remaining symptoms and domains.

## 4 Discussion

In the APC cohort of people with LC and cognitive complaints, the three most common self-reported symptoms were concentration and memory deficit, asthenia, fatigue. More than 60% presented a cognitive deficit in at least one domain, being the executive functions the most impaired. Additionally, more than half of the participants presented a dysfunctional pattern in balance; and the 9% presented a fundus retina alteration.

The demographic profile of our cohort study is similar to other studies (1, 41). According to several studies, women are more susceptible to developing LC (42, 43). Some papers propose that this may be due to a different expression of angiotensin-converting enzyme 2 (ACE-2) or transmembrane protease serine 2 (TMPRSS2) receptors, or to lower production of proinflammatory cytokines such as interleukin-6 (IL-6) in women after a viral infection (44, 45). The greater frequency of women’s participation in health-related studies may be attributed to various factors, including their tendency to care more for their health. Most people in our study had a mild or moderate clinical course of SARS-CoV-2 infection. Thus, the data from our study corroborate previous research that the morbidity associated with prolonged COVID-19 is not related to the severity of the initial infection (5, 46, 47). In our sample, the most predominant complaints were lack of concentration and memory, asthenia, fatigue, brain fog, insomnia, myalgia, vertigo and dizziness. These findings are consistent with the current literature (48, 49). It is important to consider that high percentage of health professionals in our cohort could be influence a higher detection and reporting of symptoms.

Our results show that many patients in the sample demonstrated cognitive deficits in at least one domain. This overall result supports subjective cognitive complaints with objective neuropsychological measurements. Several articles assess cognitive functioning in people with LC, and most point to lower functioning compared to healthy subjects (17, 19, 50). In our study, patients showed impairments in several cognitive domains, including executive functions, attention, speed processing, and memory. These findings are in line with recent reviews (8, 49, 51). Linguistic and visuospatial abilities appear to be more preserved, whereas memory, executive function and attention seem to be the most affected capacities in these patients (52–54). This may be because attention, memory, and executive functions are high-level cognitive processes that integrate multiple brain regions. In contrast, language and visuospatial skills are more specific modular functions that are localized to specific brain areas. Considering that, COVID-19 affects the central nervous system (CNS), several hypotheses that try to explain the cognitive impairment. The immune response induced by the SARS-CoV-2 infection resulted in inflammation of CNS through systemic chemokines and other possible mechanisms (55). Persistent elevation of cytokines, chemokines and reactive microglia in cerebrospinal fluid can dysregulate multiple neural cell types. Such as altering homeostasis and plasticity (56), impairing neurogenesis (57) and inducing neurotoxic reactivity (58), all of which can affect neural circuit function and thus cognition (59).

To our knowledge, this is the first study to examine differences in cognitive impairment in relation to the duration of cognitive symptoms such as lack of concentration and memory, brain fog, and non-specific disorientation. It should be noted that most of the comparisons were not significant, making it difficult to draw conclusions. In the domain of executive functions, it seems that more time with the symptoms (lack of concentration and memory, brain fog and non-specific disorientation) is related to greater deficits. Nevertheless, there is some dispersion in the results for the other domains. This may be because it behaves differently depending on each symptom and cognitive domain. These discrepancies may also be caused by the intervention of other factors that have not been considered, such as comorbidities, severity of LC symptoms, and cognitive reserve. Thus, the results are inconclusive; therefore, we cannot assume that the persistence of symptomatology affects the progression of cognitive deficits. According to the PHOSP-COVID research group (60), a small improvement was found at 1 year, indicating that part of this deficit was not pre-existing and is potentially modifiable; however, some persisted after 1 year in susceptible individuals. In contrast, other studies showed a lower rate of improvement after 2 years of follow-up (61).

Results of the posturography test showed a wide variety of patterns in our sample, with the most predominant being somatosensory and vestibular dysfunction. These results cannot be strictly attributed to LC due to limited evidence in the literature. Even so, Yilmaz et al. (62) proved that balance in patients undergoing COVID-19 was impaired compared to healthy individuals. The mechanisms for reduced postural control remain unclear. It is not known whether the virus causes dysfunction of the vestibular system or whether such dysfunction is the result of an infectious process within the neural structures (25). Our findings suggest that the dysfunction is not due to a specific system; but is a more generalized affectation in the different systems involved in balance. The results obtained in the study by Gervasoni et al. (63) suggest the LC balance test performances were away from normality when integrating vision, somatosensory and vestibular information. It is therefore postulated that the alterations induced by SARS-CoV-2 infection result in a failure to integrate the various sensory inputs. Nevertheless, more specific complementary tests, such as nerve conduction, nuclear resonance, sensory, and organizational tests, are required to corroborate this hypothesis.

Retinal vascular involvement following SARS-CoV-2 infection has been little studied. Nevertheless, some studies indicate that SARS-CoV-2 infection causes retinal manifestations. Vavvas et al. (64) reported that the diameter of arteries and vessels in the retina was larger in patients with COVID-19 than in healthy individuals. This could be because when the inflammatory response begins, blood supply increases and vasodilation occurs (65). Some of the fundus findings in people with recent COVID-19 infection included retinitis patches, hard exudates, cotton wool spots, and superficial hemorrhages (66, 67). In a longitudinal study conducted by Invernizzi et al. (68), they found that most of the retinal vasculature alterations regress with time after acute COVID-19. However, those who suffer from severe COVID-19 may have long-lasting retinal vessel dilation persisting. In absence of previous information, we cannot be sure that the retinal lesions are due to SARS-CoV-2. There are also no studies on the prevalence of retinal vascular lesions in the general population. Although some retinal damage has been reported in the literature, the percentage of retinal damage observed in our sample is low, suggesting that retinography may not be a sensitive instrument for detecting the type of lesions produced by SARS-CoV-2. Therefore, it may be more advisable to use other techniques such as optical coherence tomography (69).

The study’s strengths include extensive follow-up of a population with a newly established disease. Our study uses various infrequent assessments such as posturography and retinography, and extensive battery of neurocognitive tests adopting domain-specific assessment tools to provide comprehensive monitoring. Furthermore, we have endeavored to collect all the symptoms reviewed in the literature and their duration, which may aid in the delimitation of the clinical spectrum.

However, our study has several limitations. First, the limited sample size may make it difficult to find significant relationships in the data. Second, there may be a sampling bias considering that most volunteers may have wanted to participate in the study because they had considerable impairment. Third, it should be noted that the measurement of clinical symptoms depended on the participants’ recall accuracy. Lastly, the lack of a control group without LC makes it challenging to definitively attribute the observed effects to LC specifically. For this reason, future lines of research should include a control group in each clinical test. It would also be interesting to re-evaluate the same sample after some time to see the progression of the conditions.

## 5 Conclusion

This study describes retinal, balance and cognition status in individuals with LC and cognitive complaints. It provides a framework for addressing patient and family expectations regarding their anticipated health. It also provides a better understanding of the LC syndrome and facilitates awareness of the importance of clinical management in primary care. It is important to maintain and increase the sensitivity of the health system around this pathology, both at the level of health professionals and managers and the general population. Knowing the health status of these individuals can help healthcare professionals distinguish LC symptoms from pre-existing conditions, helping to formalize diagnosis and treatment. Considering that, the majority in our sample present a cognitive deficit, it is convenient to monitor the progression of cognitive deterioration. As well as implementing, a pattern of postural balance exercises as rehabilitation training for vestibular problems. From this perspective, the main objective of clinicians and researchers is to create interventions that promote cognitive stimulation and balance training. Also, that ophthalmologists or retina specialists make a proper diagnosis and, if necessary, implement a personalized treatment plan. In conclusion, it is important to follow up with these patients to control their affectations and to find an adequate multidisciplinary treatment that contemplates physical and psychological aspects.

## Data availability statement

The original contributions presented in the study are included in the article or the Supplementary material, further inquiries can be directed to the corresponding authors.

## Ethics statement

The ethics committee of the Foundation University Institute for Primary Health Care Research Jordi Gol I Gurina (IDIAPJGol) has approved the study protocol (ref. 21/220-P). This study adheres to guidelines established in the Declaration of Helsinki. All participants recruited were fully informed about study and signed informed consent to participate. They consented to use their collected data for research and agreed to the applicable regulations, privacy policies, and terms of use. Participant data has been anonymized according to a numerical coding system and stored securely in the REDCap database.

## Author contributions

MC-C: Formal analysis, Writing – original draft, Writing – review & editing, Data curation, Validation, Visualization. BL-G: Data curation, Formal analysis, Writing – original draft, Writing – review & editing, Validation, Visualization. RD-A: Writing – review & editing, Investigation, Visualization. NL-V: Writing – review & editing, Investigation, Funding acquisition, Resources, Visualization. PM-A: Writing – review & editing, Investigation, Visualization. GM: Formal analysis, Visualization, Writing – review & editing, Data curation. AA: Writing – review & editing, Investigation, Visualization. CC: Writing – review & editing, Investigation, Funding acquisition, Resources, Visualization. AC-G: Formal analysis, Writing – review & editing, Data curation, Visualization. VL-L: Writing – review & editing, Investigation, Visualization. VZ-P: Writing – review & editing, Investigation, Visualization. LL: Writing – review & editing, Methodology, Visualization. RG-S: Writing – review & editing, Methodology, Funding acquisition, Resources, Software, Visualization. AF: Writing – review & editing, Investigation, Visualization. EM-G: Writing – review & editing, Methodology, Visualization. MMas: Writing – review & editing, Funding acquisition, Project administration, Resources, Supervision, Visualization. JM-M: Writing – review & editing, Methodology, Visualization. MR-P: Writing – review & editing, Investigation, Visualization. LM: Writing – review & editing, Investigation, Funding acquisition, Resources, Visualization. AP: Writing – review & editing, Methodology, Visualization. MMat: Writing – review & editing, Methodology, Visualization. MB: Writing – review & editing, Investigation, Visualization. BQ: Writing – review & editing, Investigation, Visualization. JP: Supervision, Writing – review & editing, Funding acquisition, Project administration, Resources, Visualization. EM-C: Supervision, Writing – review & editing, Funding acquisition, Project administration, Resources, Visualization. CV: Conceptualization, Supervision, Writing – original draft, Writing – review & editing, Funding acquisition, Project administration, Resources, Visualization. PT-M: Conceptualization, Supervision, Writing – original draft, Writing – review & editing, Funding acquisition, Project administration, Resources, Visualization.

## Group member of the APC Collaborative Group (Aliança ProHEpiC-19 Cognitiu)

Aitana Ayet, Sandra Banderas, Laia Bernard, Jofre Bielsa-Pascual, Marc Boigues, Meritxell Carmona-Cervelló, Lucía A. Carrasco-Ribelles, Carla Chacón, Anna Costa-Garrido, Galadriel Diez Fadrique, Rosalía Dacosta-Aguayo, Antònia Fornés, Rosa García-Sierra, Eulàlia Grau, Noemí Lamonja-Vicente, Brenda B. León-Gómez, Liudmila Liutsko, Gemma Lladós, Cristina López, Víctor M. López-Lifante, Cora Loste, Marta Massanella, Maria Mataró, Lourdes Mateu, Eva Martínez-Cáceres, Gemma Molist, Pilar Montero-Alía, Eduard Moreno-Gabriel, Francisco Muñoz-López, Jose A. Muñoz-Moreno, Maria Nevot, Alba Pachón, Ruth Peña, Raul Pérez-Caballero, Julia G. Prado, Anna Prats, Josep Puig, Bibiana Quirant, Gabriel F. Rodriguez-Lozano, M. Carmen Rodríguez-Pérez, Sandra Sánchez-Vallespín, Jose Ramón Santos, Pere Torán-Monserrat, Macedonia Trigueros, Concepció Violán, and Valeria Zamora-Putin.
