# Supplementary material for: Long COVID: cognitive, balance, and retina manifestations
Source: Front Med (Lausanne). 2024 Jul 5;11:1399145. doi: 10.3389/fmed.2024.1399145 (PMC11260168; doi:10.3389/fmed.2024.1399145)
Supplement: Supplementary file 1 [file Data_Sheet_1.docx]

**Appendix 1.** No neurological symptoms collected.

| Cardiologic | Alteration of the heartbeat |
| --- | --- |
|  | Elevated blood pressure |
|  | Hypotension |
|  | Nonspecific bradycardia |
|  | Nonspecific chest pain |
|  | Nonspecific tachycardia |
|  | Other heartbeat abnormalities |
|  | Other types of chest pain |
|  | Palpitations |
|  | Precordial pain |
|  | Pressure lowering |
| Digestive | Abdominal distension |
|  | Change of bowel habits |
|  | Diarrhea |
|  | Heartburn |
|  | Nausea |
|  | Nausea and vomiting |
|  | Nausea without vomiting |
|  | Nonspecific vomiting |
|  | Pelvic and abdominal pain |
|  | Vomiting without nausea |
| General | Abnormal weight loss |
|  | Anorexia |
|  | Asthenia |
|  | Chronic fatigue syndrome |
|  | Discomfort and fatigue |
|  | Disturbance of autonomic nervous system |
|  | Other types of fatigue |
|  | Other types of malaise |
|  | Unspecified fever |
|  | Unspecified visual disturbances |
| Hormonal | Menstrual cycle alterations |
|  | Other hormonal changes |
| Ocular | Bilateral dry eye syndrome |
|  | Conjunctivitis |
|  | Nonspecific dry eye syndrome |
| Otorhinolaryngology | Acute pharyngitis |
|  | Anosmia |
|  | Aphonia |
|  | Chronic sinusitis |
|  | Dysphonia |
|  | Epistaxis |
|  | Olfactory alteration taste |
|  | Other nasal and sinus |
|  | Other olfactory and taste alteration |
|  | Parageusia |
|  | Pharyngeal pain |
|  | Tinnitus |
|  | Vasomotor rhinitis |
|  | Vestibular disorder |
| Pulmonary | Acute interstitial pneumonitis |
|  | Chest pain on respiration |
|  | Cough |
|  | Idiopathic pulmonary fibrosis |
|  | Other forms of dyspnea |
|  | Other interstitial lung disease |
|  | Other interstitial lung diseases with fibrosis |
|  | Other specified interstitial lung diseases |
|  | Pulmonary embolism |
|  | Unspecified interstitial lung disease |
|  | Unspecific pulmonary fibrosis |
|  | Unspecific dyspnea |
|  | Wheezing |
| Rheumatic | Myalgia |
|  | Unspecified arthralgia |
| Urologic | Bladder urgency |
|  | Dysuria |
|  | Nonspecific painful urination |
|  | Polyuria |
|  | Voiding pain |
| Skin | Allergic dermatitis |
|  | Alopecia areata |
|  | Androgenic alopecia |
|  | Atopic dermatitis |
|  | Dry syndrome |
|  | Exanthema and others |
|  | Exfoliative dermatitis |
|  | Idiopathic urticaria |
|  | Irritant dermatitis |
|  | Nonspecific alopecia |
|  | Nonspecific atopic dermatitis |
|  | Nonspecific contact dermatitis |
|  | Nonspecific dermatitis |
|  | Nonspecific serostomy |
|  | Nonspecific urticaria |
|  | Oral aphthous ulcers |
|  | Other alopecia |
|  | Other dermatitis |
|  | Other types of dermatitis |
|  | Other urticaria |
|  | Pruija |
|  | Seborrheic dermatitis |

**Appendix 2.** Expert clinical consensus established for balance patterns based on Romberg’s test indices.

| Patterns type | Somatosensory | Vestibular | Visual |
| --- | --- | --- | --- |
| Normal or Compensated | ≥ 95% | ≥ 95% | ≥ 95% |
| Somatosensory dysfunction | < 95% | ≥ 95% | ≥ 95% |
| Vestibular dysfunction | ≥ 95% | < 95% | ≥ 95% |
| Visual dysfunction | ≥ 95% | ≥ 95% | < 95% |
| Multi-sensory dysfunction | < 95% | < 95% | < 95% |
| Somatosensory dependence | ≥ 95% | < 95% | < 95% |
| Vestibular dependence | < 95% | ≥ 95% | < 95% |
| Visual dependence | < 95% | < 95% | ≥ 95% |

**Appendix 3.** No neurological symptoms self-reported at time of assessment (n=166).

|  | Total | |
| --- | --- | --- |
| No neurological symptoms | n | (%) |
| **Cardiologic** | **76** | **(46.34)** |
| Alteration of the heartbeat | 10 | (13.16) |
| Elevated blood pressure | 6 | (7.89) |
| Hypotension | 3 | (3.95) |
| Nonspecific bradycardia | 9 | (11.84) |
| Nonspecific chest pain | 15 | (19.74) |
| Nonspecific tachycardia | 51 | (67.11) |
| Other types of chest pain | 1 | (1.32) |
| Palpitations | 15 | (19.74) |
| Precordial pain | 13 | (17.11) |
| Pressure lowering | 3 | (3.95) |
| **Digestive** | **96** | **(58.90)** |
| Abdominal distension | 65 | (67.71) |
| Change of bowel habits | 60 | (62.50) |
| Diarrhea | 66 | (68.75) |
| Heartburn | 53 | (55.21) |
| Nausea | 33 | (34.38) |
| Nausea and vomiting | 23 | (23.96) |
| Nonspecific vomiting | 1 | (1.04) |
| Pelvic and abdominal pain | 62 | (64.58) |
| **General** | **152** | **(92.68)** |
| Abnormal weight loss | 44 | (28.95) |
| Anorexia | 30 | (19.74) |
| Asthenia | 143 | (94.08) |
| Chronic fatigue syndrome | 4 | (2.63) |
| Discomfort and fatigue | 138 | (90.79) |
| Disturbance of autonomic nervous system | 1 | (0.66) |
| Other types of fatigue | 3 | (1.97) |
| Unspecified fever | 30 | (19.74) |
| Unspecified visual disturbances | 41 | (26.97) |
| **Hormonal** | **42** | **(25.61)** |
| Menstrual cycle alterations | 11 | (26.19) |
| Other hormonal changes | 3 | (7.14) |
| **Ocular** | **61** | **(37.42)** |
| Bilateral dry eye syndrome | 43 | (70.49) |
| Conjunctivitis | 17 | (27.87) |
| Nonspecific dry eye syndrome | 2 | (3.28) |
| **ORL** | **100** | **(61.35)** |
| Acute pharyngitis | 6 | (6.00) |
| Anosmia | 54 | (54.00) |
| Aphonia | 24 | (24.00) |
| Chronic sinusitis | 2 | (2.00) |
| Dysphonia | 8 | (8.00) |
| Epistaxis | 8 | (8.00) |
| Olfactory alteration taste | 10 | (10.00) |
| Parageusia | 53 | (53.00) |
| Pharyngeal pain | 8 | (8.00) |
| Tinnitus | 43 | (43.00) |
| Vasomotor rhinitis | 3 | (3.00) |
| **Pulmonary** | **86** | **(52.76)** |
| Acute interstitial pneumonitis | 2 | (2.33) |
| Chest pain on respiration | 37 | (43.02) |
| Cough | 47 | (54.65) |
| Idiopathic pulmonary fibrosis | 1 | (1.16) |
| Pulmonary embolism | 2 | (2.33) |
| Unspecified interstitial lung disease | 1 | (1.16) |
| Unspecific pulmonary fibrosis | 3 | (3.49) |
| Unspecific dyspnea | 77 | (89.53) |
| Wheezing | 12 | (13.95) |
| **Rheumatic** | **122** | **(74.39)** |
| Myalgia | 115 | (94.26) |
| Unspecified arthralgia | 114 | (93.44) |
| **Urologic** | **33** | **(20.37)** |
| Bladder urgency | 6 | (18.18) |
| Dysuria | 2 | (6.06) |
| Nonspecific painful urination | 3 | (9.09) |
| Polyuria | 13 | (39.39) |
| Voiding pain | 13 | (39.39) |
| **Skin** | **70** | **(45.75)** |
| Alopecia areata | 11 | (15.71) |
| Atopic dermatitis | 1 | (1.43) |
| Dry syndrome | 32 | (45.71) |
| Exanthema and others | 2 | (2.86) |
| Exfoliative dermatitis | 1 | (1.43) |
| Idiopathic urticaria | 5 | (7.14) |
| Nonspecific alopecia | 17 | (24.29) |
| Nonspecific dermatitis | 35 | (50.00) |
| Nonspecific serostomy | 32 | (45.71) |
| Nonspecific urticaria | 5 | (7.14) |
| Oral aphthous ulcers | 12 | (17.14) |
| Other alopecia | 8 | (11.43) |
| Other dermatitis | 2 | (2.86) |
| Pruija | 37 | (52.86) |

ORL, Otorhinolaryngology.

Note: The no neurological symptoms self-reported had several missing values.

**Appendix 4.** Demographic and clinical characteristics of the two groups according the duration of the symptoms.

|  | G1  (n = 90; 54.88%) | G2  (n = 74; 45.12%) | χ² | p |
| --- | --- | --- | --- | --- |
| Variable | n (%) | n (%) |  |  |
| **Gender** |  |  |  |  |
| Female | 71 (78.89) | 62 (82.67) | 0.37 | 0.541 |
| Male | 19 (21.11) | 13 (17.33) |  |  |
| **Job field** |  |  |  |  |
| Medical doctor | 8 (8.89) | 2 (2.70) | 9.51 | 0.090 |
| Nurse | 18 (20.00) | 10 (13.51) |  |  |
| Health services | 8 (8.89) | 2 (2.70) |  |  |
| Health assistant | 8 (8.89) | 9 (12.16) |  |  |
| Others | 48 (53.33) | 51 (68.92) |  |  |
| **Vascular Risk** |  |  |  |  |
| Hypertension | 19 (21.11) | 14 (18.92) | 0.15 | 0.696 |
| High Cholesterol | 20 (22.22) | 18 (24.32) | 0.22 | 0.640 |
| Diabetes | 4 (4.44) | 1 (1.35) | 1.35 | 0.246 |
| Alcohol | 31 (34.44) | 30 (41.10) | 0.65 | 0.422 |
| Smoking^a^ | 40 (44.95) | 35 (47.3) | 1.07 | 0.585 |
| **Clinical spectrum COVID-19**^b^ |  |  |  |  |
| Asymptomatic | 2 (2.22) | 0 (0) | 2.52 | 0.472 |
| Mild-Moderate | 66 (73.33) | 58 (78.38) |  |  |
| Hospitalization | 19 (21.11) | 15 (20.27) |  |  |
| ICU | 3 (3.33) | 1 (1.35) |  |  |
|  | Mean [SD] | Mean [SD] | t | p |
| **Age** (years) | 48.66 [8.45] | 50.10 [8.34] | -1.12 | 0.267 |
| **Education** (years) | 13.94 [3.28] | 13.56 [2.48] | 0.84 | 0.405 |
| **BMI** | 28.08 [6.59] | 27.59 [6.35] | 0.55 | 0.583 |
| **Times diagnostic COVID-19** | 1.58 [0.64] | 1.78 [0.93] | -1.72 | 0.088 |

M, Mean. SD, Standard Deviation. BMI, Body Mass Index. ICU, Intensive Care Unit.

Note: Symptom duration was divided into two groups: G1 (1 to 25 months) and G2 (26 to 36 months).

^a^ The smoking category includes smokers and ex-smokers.

^b^ Clinical spectrum variable refers to the first time of SARS-CoV-2 infection.
